# Recruiting hard-to-engage groups to online psychosocial interventions: Experiences from an RCT study targeting adolescents with a visible difference

**DOI:** 10.1016/j.conctc.2021.100869

**Published:** 2021-11-18

**Authors:** Johanna Kling, Tine Nordgreen, Ingela L. Kvalem, Heidi Williamson, Kristin B. Feragen

**Affiliations:** a Centre for Rare Disorders, Rikshospitalet, Oslo University Hospital HF, Norway; b Haukeland University Hospital, Bergen, Norway; c Department of Psychology, University of Oslo, Norway; d Centre for Appearance Research, University of the West of England, Bristol, UK

**Keywords:** Randomised control trial, Participant recruitment, Adolescents, Difficult-to-engage, Online intervention

## Abstract

Online interventions have the potential to reach individuals who are otherwise difficult to engage due to stigma and sensitive topics. However, these individuals also tend to be hard to recruit in clinical trials, a crucial step in order to provide evidence-based interventions. This highlights a need for more information about efficient recruitment strategies for difficult-to-engage groups. The present study aimed to share the systematised experiences of recruiting adolescents with a visible difference to an online psychosocial intervention RCT. With the intention to recruit 160 participants (age 12–17), recruitment efforts were nationwide and included multiple arenas (e.g., hospitals, schools, social media), and methods (e.g., in-consultation, targeted letters, posters). Ultimately, 102 participants were recruited, and results showed that recruitment involving patient organisations, hospital departments, and specialised resource centres were most successful in reaching participants. The most efficient recruitment strategy was targeted letters sent home to eligible patients/members, as 78% of the participants were recruited this way. Media and social media recruitment efforts yielded comparatively few participants. No participants were recruited through schools and educational health care services, primary health care services, or municipal and regional authorities. Our results are discussed in relation to barriers with recruiting difficult-to-engage groups to RCTs, providing useful recruitment tools to future similar studies. For instance, future studies are recommended to utilise targeted approaches over general population approaches. Also, results from recruitment efforts should routinely be reported, as this ultimately will provide more general strategies for effective recruitment and support studies in reaching recruitment goals.

## Highlights

• Studies about efficient trial recruitment for difficult-to-engage groups are lacking
• Adolescents with a visible difference tend to be difficult to engage in research
• Successful recruitment include patient organisations and specialised units
• The most efficient recruitment method is targeted letters
• Results from recruitment efforts should routinely be reported

## 1 Introduction

Online psychosocial interventions have the potential to reach individuals who are otherwise difficult to engage due to sensitive topics and fear of stigma, e.g., adolescents with a visible difference. Visible difference, i.e., conditions or injuries that alter or affect a person's appearance, can cause psychological and social struggles [1,2]. Although some adolescents adjust positively to living with a visible difference, long-term negative outcomes related to socialising, self-perceptions, or body image are common [3,4]. Conducting psychosocial intervention research on adolescents with visible differences is a growing area, and previous studies have highlighted the lack of high-quality evidence-based interventions [5]. However, recruitment to intervention studies, e.g., randomised control trials (RCTs), tends to be challenging and there is a knowledge gap with regard to effective strategies aimed at those recruiting to trials [6,7]. In particular, research studies sharing experiences with various recruitment channels and strategies for hard-to-engage groups are lacking.

Common barriers in intervention recruitment, especially when targeting adolescents, include fear of disclosure of personal information and potential peer stigma, psychosocial stressors (e.g., being reminded about one's difficulties), and health care providers' general (in)ability to prioritise the research (e.g., [[8], [9], [10], [11], [12]]. Moreover, although online interventions provide unique opportunities (e.g., reach), they also demand increased individual responsibility and motivation for their completion [13]. Nonetheless, adolescents' acceptability for online psychosocial interventions is generally good (e.g., [14,15].

In addition to general barriers, engagement specifically in appearance research is often experienced as sensitive and confronting [16]. Health professionals and parents tend to have appearance misconceptions (e.g., believing that most psychosocial problems will be solved with surgery), or experience the topic as sensitive, and are thus reluctant to discuss interventions with an adolescent [[17], [18], [19], [20]]. Recruiting boys to appearance-related interventions has also previously been described as especially difficult [16].

General recommendations for RCT planning comprise identifying and engaging stakeholders (e.g., health care providers), identifying where potential participants seek information, and utilising appropriate channels [6,7,21]. To overcome barriers specifically associated with recruiting adolescents with visible difference, complementary recruitment strategies have been suggested [15,16]. Importantly, it has been proposed that researchers should utilise multiple recruitment strategies. Recruitment should not rely solely on in-consultation recruitment, since this method is time consuming, biased by the health professionals’ attitudes towards appearance research, and can be experienced as overwhelming for patients [17]. Other suggestions are to maximise reach through social media and by utilising hospital media and communication departments, newsletters, and e-mail networks [16].

Recruiting difficult-to-engage groups to psychosocial intervention studies tends to be difficult [12,16], and research describing systematised recruitment efforts is lacking [6,7]. By synthesising and detailing our experiences of recruiting young people with a visible difference to the [Norway] Young Persons’ Face IT study, the aim of this paper is to share (un)successful recruitment channels and strategies that may benefit future intervention studies targeting other hard-to-engage groups.

## 2 Method

### 2.1 The young persons’ face IT online intervention

The original YP Face IT online intervention was developed in the UK and in close collaboration with young people and clinical experts [22], modelled on a similar successful and evidence-based programme aimed at adults with a visible difference (i.e., Face IT; [23]. YP Face IT includes seven sessions (+booster session), and is an online intervention aimed to provide psychosocial support to young people (age 12–17) with a visible difference. Specifically, the aim of the intervention is to reduce appearance-related distress and social anxiety, thereby strengthening psychological adjustment to a congenital or acquired visible difference. The intervention provides easy access to specialist advice and support via a home computer/tablet, using illustrations, information, videos, interactive activities, and a discussion forum for participants only (supervised by the research team). Through these tools, YP Face IT provides advice and teaches coping skills based on cognitive behavioural therapy and social interaction skills training, intervention approaches that have shown promise within this specific field of research [[24], [25], [26]]. In the UK, the YP Face IT has been evaluated with good results regarding feasibility and acceptability, with no intervention-related adverse effects reported [15,22]. YP Face IT has also been piloted in the US [27], and is currently under trial in the Netherlands [28] and [Country] [29].

#### 2.1.1 The YP face IT-[Country] pilot study

Before the YP Face IT-[Country] RCT study was initiated, a [Country] YP Face IT pilot RCT study was carried out [30]. The pilot study supported the project's acceptability and feasibility in [Country], based on a sample of 29 young people with a visible difference [30]. Experiences from the pilot study were used to refine plans for the larger RCT study and calculate sample size, based on experiences with attrition and estimated participation rate. As an example, the pilot revealed that some parents struggled to understand the need for a control group, and informed the research team that the risk of not being randomised to the intervention group reduced their own or their adolescent's motivation for participation. Hence, in order to improve recruitment, reduce ethical considerations, and handle potential disappointment for those who were randomised to the control group, a waitlist control group design was imposed for the larger RCT study, as also suggested by the British feasibility study [15]. Moreover, the [Country] pilot study also showed that it was hard to recruit participants from primary care settings [30].

### 2.2 Design

The YP Face IT-[Country] RCT is funded by the Research Council of [Country], reviewed by the Regional Committee for Medical Research Ethics ([Health region], reference number: [Number]) and accepted by the Data Protection Office based at [City] University Hospital. The study is a 4-year study (2019–2023).

Based on previously described experiences from the pilot study, two important changes were made to the study protocol for the RCT: (1) more focus on recruitment via specialist health care settings over primary care settings, and (2) utilise a waitlist control group with opportunity to receive the intervention after three months. Recruitment was planned from April 2019 to June 2020, but due to lower recruitment rates than hypothesised, this period was extended to February 2021 and resulted in the final inclusion of 102 participants to the YP Face IT-[Country] RCT. The research team in charge of recruitment consisted of the first and last authors, a PhD candidate, and two research assistants.

#### 2.2.1 Participants and procedure

National statistics currently indicate there are approximately 400 000 adolescents aged 12–17 in [Country] [31]. Estimates suggest that approximately 2% have a visible facial or bodily difference that deviates from the norm [32], which means that around 8000 young people aged 12 to 17 could possibly be relevant for inclusion. Based on estimates of study power and retention, we aimed to recruit 160 young people with any appearance-altering condition, injury, or treatment side-effect, who also self-identified as experiencing appearance-related distress, teasing, or bullying. This number was considered reachable given the total number of potential participants. Young people interested in participating contacted the research team and were screened for eligibility by a research team member via telephone. For potential participants under the age of 16, parents were also contacted. Specific inclusion criteria were (1) age 12–17 with an appearance-altering condition, and experiencing appearance-related distress, teasing, or bullying, (2) access to a home computer/tablet and internet; (3) reading level >12 years of age (audio recordings for all written text available on the website for those who may struggle with reading), and (4) normal/corrected-to-normal vision. Exclusion criteria included (1) diagnosed clinical depression, psychosis, and/or eating disorder (alternative support necessary), (2) post-traumatic stress disorder (PTSD) or within 12 months of traumatic injury (alternative support necessary), (3) learning disability that would impede understanding of the programme's content, and (4) currently receiving psychological face-to-face interventions.

In total, 137 potential participants contacted the research team and were screened for inclusion and exclusion criteria via telephone. Ultimately, 102 participants were included in the study, six did not meet inclusion/exclusion criteria and 29 changed their minds/did not respond back to the research team after screening. Participants’ age ranged between 11 and 18 (*M* = 13.9*; SD* = 1.7), 58% were girls, and visible differences included e.g., craniofacial conditions, scarring, differences in body form, or skin conditions. Incentives were used; all participants received multi-use gift cards after completing follow-up questionnaires.

#### 2.2.2 Recruitment channels and strategies

Recruitment was broad, nationwide, and inclusive of multiple sources and methods. In addition to the YP Face IT-[Country] programme website (ungfaceit.no), the YP Face IT-[Country] research team created an informational webpage (ungfaceit.info) and a Facebook-page in order to raise awareness and spread information about the project and the intervention on social media. Contacts were re-established with the organisations, specialised treatment centres, and hospital departments that contributed with recruitment to the pilot study, and additional relevant stakeholders were contacted and engaged [6]. Members of the pilot study's Advisory Group (AG) agreed to be part of the AG for the larger RCT study. An updated and broader search for relevant patient organisations was performed, and new members included. The larger RCT AG group finally consisted of representatives (adults and young adults) from 18 different patient organisations (representing people with different conditions, such as craniofacial conditions, skin conditions, short stature, overweight, and burns).

Patient organisations, schools and educational health care services, specialised resource centres, hospital departments, municipal and regional authorities were first approached by informational e-mails, which were followed up by phone calls from a research assistant or PhD student. They were informed about the project and asked to help with the recruitment (e.g., through in-consultation recruitment, targeted letters to patients/members, sharing information on websites/social media/member magazines, and/or hanging up posters and spreading brochures/flyers). Based on experiences from the pilot study [30], more effort (e.g., additional e-mails, phone calls, and personal contact) was given to establishing contact with hospital departments and specialised resource centres, over primary health care settings. Recruitment strategies were continuously and regularly reviewed, so that efforts could be tailored to our on-going experiences with recruitment. Hence, recruitment followed pragmatic considerations, i.e., in relation to the overarching aim of conducting an RCT it was considered more important to recruit as many participants as possible, rather than to put equal effort into every strategy. In addition, the project was presented nationwide and face-to-face to interested hospital departments, specialist care units, patient organisations, as well as at conferences, in order to boost recruitment interest. Popular people on [Country] social media (i.e., influencers/YouTubers with and without visible difference) were invited to promote the project and the YP Face IT programme through different social media channels in exchange for economic compensation. The project was also presented through participation on national news and TV (news and morning show). An article about the project was also published on a national public information channel for young people in [Country] (ung.no).

Based on experiences from the pilot study [30], it was estimated that the project would recruit approximately the following number of participants through different channels: targeted letters sent from specialised treatment units/hospital departments (*n* = 60), primary care (*n* = 60), patient organisations (*n* = 20), and media/social media strategies (*n* = 20), resulting in a total of 160 participants or more. Although experiences from the pilot study indicated that recruitment from primary care settings was not as successful as anticipated, 60 participants was considered an appropriate estimate as there are far more primary care settings than specialised settings, with more consistent reach in the population.

#### 2.2.3 Recruitment data

The data displayed in our paper are descriptive and based on recruitment information from the YP Face IT-[Country] project. From the start of the project, all contacts with patient organisations, schools and educational health care services, primary health care services, specialised resource centres, hospital departments, municipal and regional authorities/instances (e.g., county directors of public health and municipal health care directors), and individuals (e.g., social media influencers) were thoroughly recorded by the project team. A joint document was created that contained information regarding who was contacted, in what way and when, along with their response to participate in recruitment efforts (acceptance, declination, or non-response). If accepting, information regarding recruitment method and estimated/definite reach was noted and recurring follow-ups were done to ensure accuracy in the reporting. During their first screening to YP Face IT-[Country], participants were asked where they had received information about the project (i.e., from whom and in what way). This information was compiled with the recruitment information from the document and randomisation information from the YP Face IT-[Country] program, to create Table 1 displayed in the result section.

**Table 1:** Recruitment data in terms of type of recruitment channel, methods of recruitment, estimated reach and number of adolescents participating in first screening and included (i.e. randomised to intervention group or control group) in the YP Face IT-[Country] trial, and inclusion rates.

Patient organisations (contacted *n* = 28; contributed to recruitment *n* = 16)				
Recruitment method	No of organisations (using this method)	Estimated reach	First participant screening	Included in study
Targeted letters	3	89	9	8
Targeted e-mails	5	340	6	2
Facebook (public)	9	∼18 000	0	–
Facebook (member)	5	∼4500	7^a^	4
Website	5	–	2	1
Member magazine	3	–	1	–
At meeting or via other member	–	–	9^b^	8.5^c^
Participants recruited through patient organisations: *n* = 23.5 (inclusion rate 69%)				
**Schools and Educational health care services** (contacted *n* = 87; contributed to recruitment *n* = 7)				
Recruitment method	No of EHCS (using this method)	Estimated reach	First participant screening	Included in study
Informing network	2	–	0	–
Posters and brochures	6	–	0	–
Participants recruited through schools and educational health care services *n* = 0				
**Primary health care services (incl. youth clinics)** (contacted *n* = 119; contributed to recruitment *n* = 19)				
Recruitment method	No of GP's (using this method)	Estimated reach	First participant screening	Included in study
Posters and brochures	19	–	0	–
Participants recruited through primary health care services: *n* = 0				
**Specialised resource centres** (contacted *n* = 11; contributed to recruitment *n* = 5)				
Recruitment method	No of units (using this method)	Estimated reach	First participant screening	Included in study
Targeted letters	2	944	65^d^^,^^e^	53^c^
Posters and brochures	4	–	0	–
In-consultation	–	–	2	1
Facebook (public)	1	∼2000	1	1
Website	1	–	0	–
Participants recruited through specialised resource centres; *n* = 55 (inclusion rate 81%)				
**Hospital departments (at 18 hospitals nationwide)** (contacted *n* = 231; contributed to recruitment *n* = 36)				
Recruitment method	No of departments (using this method)	Estimated reach	First participant screening	Included in study
Targeted letters	1	200 + 100^f^	19	19
Posters and brochures	20	–	5^e^	2.5^c^
Informing network	15	–	–	–
In-consultation	3	–	2^d^	0.5^c^
Website	1	–	–	–
Participants recruited through hospital departments: *n* = 22 (inclusion rate 85%)				
**Municipal and regional authorities** (contacted *n* = 66; contributed to recruitment *n* = 7)				
Recruitment method	No of departments (using this method)	Estimated reach	First participant screening	Included in study
Informing network	6	–	–	–
Posters and brochures	1	–	0	–
Participants recruited through municipal and regional instances: *n* = 0				
**Recruitment through media and social media**				
Recruitment method	Content	Estimated reach	First participant screening	Included in study
Social media	«Helsesista» promoting YP Face IT-[Country], one video posted one time in five social media channels (i.e., Snapchat, Instagram, YouTube, Facebook, Twitter).	200 000 subscribers^g^	4	1
Social media	«Daniel og Simen» promoting YP Face IT-[Country], one video posted one time on YouTube.	58 500 subscribers^g^	2^b^	0.5^c^
National TV (news)	Reportage about YP Face IT-[Country] and a participant	–	0	–
National TV (morning show)	Interview with YP Face IT-[Country]'s project leader and a young person with visible difference	–	1	0
National news ([website address])	Reportage about visible difference with YP Face IT-[Country]'s project leader	–	0	–
Official national youth's website (ung.no)	Article about YP Face IT-[Country]	–	0	–
Participants recruited through media and social media: *n* = 1.5 (inclusion rate 21%)				
**Total number of participants recruited and included in the RCT:***n* = 102 (inclusion rate 74%)				

a One participant also received information from a patient organisation website. Two participants also received a targeted e-mail from a patient organisation.

b One participant reported receiving information from both a patient organisation (via other member**)** and YouTube (Daniel og Simen).

c Participants reporting receiving information from two different sources (e.g., patient organisation *and* media) are counted as 0.5 for each in the inclusion rates.

d One participant reported receiving information from targeted letter from a specialised resource centre *and* from in-consultation at hospital department.

e Five participants reported receiving information from targeted letter from a specialised resource centre *and* a poster at a hospital department.

f Targeted letters were sent out at two occasions with a large overlap in patients receiving the letters between occasion one and two.

g Numbers retrieved from own website (helsesista.no) and YouTube channel (youtube.com/channel/UCp-DIi5oNk5WZWklxAUiLOg), November 2020.

## 3 Results

Table 1 displays the synthesised recruitment data. It shows which and how many potential recruitment channels (and individuals) were approached and asked to contribute with recruitment, and how many of these accepted. For the recruitment contributors, the table further displays methods of recruitment and number of contributors using each method, estimated reach, and the number of young persons who were screened for eligibility subsequently included in the YP Face IT-[Country] trial.

Overall, the results show that the most successful way of recruiting participants to the study was via patient organisations, hospital departments, and specialised resource centres. The most efficient method used by these three channels were recruitment through targeted letters sent home to age eligible participants registered as patients/members. The letters contained information about the study from the YP Face IT-[Country] team, as well as a cover page from the current organisation/department/centre. In total, this method resulted in 78% (*n* = 80) of the participants included in the study. For patient organisations, recruitment through word-of-mouth information (e.g., at member meetings) resulted in eight participants, which was the same number of participants that were reached by sending targeted letters to eligible members.

Recruitment via schools and educational health care services, primary health care services (incl. youth clinics), and municipal and regional authorities/instances was unsuccessful. Few schools and educational health care services wanted to participate in recruitment, most did not respond, or responded that they would not be able to reach the population under study. Recruitment through media (by social media influencers and through participation in national TV) resulted in seven people contacting the research team for participation, but five of these were then excluded due to study eligibility criteria, and one had also received information from a patient organisation.

Nine participants reported receiving information about the project from two sources, and the most common combination was being sent a targeted letter from a specialised treatment unit and viewing a poster at a hospital department.

## 4 Discussion

With the intention to display more and less successful recruitment channels and strategies for future intervention studies targeting hard-to-engage groups, the aim of our study was to synthesise and share the experiences of recruiting young people with a visible difference to the [Country] Young Persons’ Face IT study. Overall, results showed that the most successful recruitment route was via targeted letters sent home to potential participants by patient organisations, hospital departments, or specialised resource centres. At study screening, 93% (*n =* 128) of the participants reported receiving information about the study from one of these three recruitment contributors, and 68% (*n* = 93) reported receiving targeted letters. Inclusion rates (i.e., number of participants included in the study after screening) were highest for participants recruited via hospital departments and specialised resource centres: 85% and 81%, respectively. These results have several implications.

First, our results suggest that eligible adolescents are reached when using specialised recruitment channels, in contrast to when recruitment efforts are more general and channelled through primary care. This is in line with previous studies demonstrating that young people infrequently consult in primary care, and therefore can be hard to reach through this channel [17]. The advantage of engaging potential participants through specialised units is that they are easily identified and reached. The drawback is that young people who self-identify as visibly different, but who are not members of any patient organisation and/or followed-up by specialised treatment settings, will not be reached this way. Given that distress levels vary irrespective of physical characteristics such as cause, location, and severity of the visible difference [33], future recruitment procedures need to ensure that as many eligible participants as possible are reached, regardless of whether they are or are not under medical follow-up. These considerations are most likely applicable to other hard-to-engage groups and sensitive topics as well (e.g., relating to appearance concerns, sexuality, addiction etc.), were there are individuals who would benefit from support, but that are not formally registered as patients or members. However, recruitment of hard-to-engage groups through primary care settings should not be dismissed as a recruitment method. Although the present study did not manage to recruit participants via primary care settings, there are examples of previous studies that have been successful. For instance [17], successfully recruited participants to another YP Face IT study by sending out invitation letters to all young people on primary care patients lists, signed by the patients’ GP to add credibility [17].

Second, when information about a study is provided through specialised treatment units, an established level of rapport and trust in the treatment team may increase response rates [16], as seemed evident in the present results. The same effect may explain higher recruitment rates when information is provided through a patient organisation. It is also noteworthy that the one hospital department most successful with participant recruitment (by sending out targeted letters at two occasions) was a department with a national responsibility for treatment of children with craniofacial conditions, which again highlights the importance of identifying relevant stakeholders in RCT planning [6].

Third, targeted letters were sent to the young person's primary caregivers for those under the age of 16. For the older adolescents, the letter was sent to the adolescent's home address (i.e., likely also the primary caregiver's address), which in both cases meant that parents generally were aware of the study. Hence, targeted letters as recruitment method could provide an opportunity for the family to discuss participation at home, with more privacy, and without time pressure or what could be experienced as expectations from health professionals [16]. Hence, the recruitment strategy of sending targeted letters to eligible participant's home addresses is likely to be generalised to other hard-to-engage groups and settings were face-to-face recruitment might be possibly experienced as overwhelming to patients.

Utilising media and social media (e.g., newsletters, paid advertisements, free posts to relevant groups/pages) has previously been acknowledged as successful strategies in psychosocial RCT recruitment, and also been suggested as potential means to maximise reach, specifically in research involving adolescents with a visible difference [16,34]. However, in the present project, the anticipated number of participants (*n* = 20) to be recruited through media and social media was overestimate. Only seven participants were reached in this way, and five of these were subsequently excluded after first screening, indicating that these approaches did not reach the target population. In addition to articles about the project within National news papers and TV news programmes, we engaged three social media influencers, two (working together on a joint YouTube channel) with a visible difference and one without a visible difference. In collaboration with the research team, they curated and posted their own video about the study on popular social media platforms, (e.g., YouTube, Snapchat, Instagram). However, the influencers only posted their videos once on each platform. Repeated posts and asking others to share the videos may have yielded a greater response from possible participants. Although influencers have been found to be able to increase positive attitudes about health-care (e.g. [35], further investigation is required to explore whether and how social media influencers can best be involved to encourage study participation. For instance, the potential influence of one versus several social media posts in participant recruitment could be explored in future studies. It is also noteworthy that patient organisations recruiting through social media (i.e., Facebook) resulted in some eligible participants. Hence, social media should not be underestimated as a recruitment strategy, but, as has been stated elsewhere [36], the success of social media recruitment seems to be very much linked to type of posts and specific platforms.

Although 102 adolescents were included in the YP Face IT-[Country] trial, we were unable to reach our goal of 160 participants. Moreover, the recruitment phase was, in line with many other studies [6], substantially prolonged. It should be acknowledged that recruitment took place during the Covid-19 pandemic, which might have influenced recruitment, for instance by affecting stakeholders and participants’ ability to prioritise the project. However, since recruitment started before the pandemic and was slow from start, the pandemic does not fully explain why engaging young people in this study was challenging. Our recruitment experiences align with previous similar projects that targeted adolescents with a visible difference (e.g. [16,27,28], who also reported similar difficulties around engaging stakeholders (e.g., health care personnel), exceeding the planned time for recruitment, and not reaching recruitment goals. Several possible explanations other than those already mentioned above may account for this shared experience.

It is well-known from previous visible difference research that appearance is considered a sensitive topic and both parents and health care providers often feel insecure about raising the topic of potential appearance issues [17,18,20]. As recommended by [16]; future studies should further investigate individual, as well as collective, barriers within hospital and care settings that might hinder health professionals from raising the topic and subsequently addressing appearance concerns, and promoting research trials such as the YP Face IT-[Country]. From an adolescent perspective, engaging in a psychosocial intervention focused on appearance concerns can be challenging as by doing so, the adolescent has to acknowledge they look different, which can increase their vulnerability during a period where much focus is on fitting in [20]. On a positive note, although previous research has described recruiting boys to appearance-related interventions as a general difficulty (for example [16], 42% of participants in our study were boys. This indicates that our recruitment strategies were successful in reaching both girls and boys.

The present study should be viewed in light of its limitations. We did not include retention rates, which might have provided information about drop-out rates in relation to recruitment strategy. In order to more comprehensively explore the usefulness of different recruitment strategies, future similar studies should consider including this information. Although some participants reported receiving information about the study from multiple sources, we did not systematically ask about this at screening. Hence, there are probably more participants receiving information through different channels but only reporting one. Another limitation concerns the fact that the different recruitment strategies did not receive equal amounts of time or energy, which in turn probably influenced which strategy appeared more or less successful. For instance, since we knew that schools tend to be inundated by research proposals and typically decline participation, we only sent out single invitation e-mails with no follow up to non-responders. This was done for pragmatic reasons (e.g., when over 50 e-mails had been sent to schools, leading to only one response, this method was quickly abandoned in favour of other strategies with more visible and less effortful results), since the aim of recruiting participants to the RCT was considered more important than using stringent recruitment strategies, and the present study was designed in relation to the overarching aim of conducting the RCT. However, in order to more systematically compare different approaches, future studies could be tailored specifically to investigate effects of different recruitment channels and strategies for hard-to-engage groups.

Moreover, as many of the recruitment issues found in our study also have been acknowledged in previous YP Face IT studies (e.g. [27,28], the question of whether YP Face IT in its current format is the most acceptable way to support adolescents with a visible difference might be justified. Future studies need to further investigate potential programme-specific barriers in order to answer this question. For instance, and preferably by engaging adolescents with a visible difference in the process, by converting the programme into an accessible mobile app instead of a web-based programme, or engaging parental involvement, could be explored.

It is also important to highlight that the experiences described in the present study are from one single study, carried out in [country], therefore not all results may be generalisable to other settings. However, since most previous studies of recruitment methods involve hypothetical trials with unknown applicability of their results to the real world [7], an important strength of our study is the thorough registration of the recruitment methods and procedures carried out in a real-world context, as well as the publication of our experiences. We encourage future researchers conducting pilot or full RCTs that targets hard-to-engage groups to also record and share both their positive and less successful recruitment efforts. Taken together, these experiences can provide a more comprehensive overview of strategies for effective recruitment.

To conclude, in addition to previous recommendations for careful recruitment planning (e.g. [6], future RCTs involving adolescents and targeting sensitive topics such as visible difference, are encouraged to utilise multi-stakeholder and targeted approaches over general population approaches, e.g., by actively involve a wide range of organisations, hospital departments, and treatment centres that can send out targeted and tailored study information to their patients or members. Future studies are also encouraged to routinely report on their recruitment efforts as, ultimately, these efforts will help to reach and favour difficult-to-engage groups in need of psychosocial support.

## Declaration of competing interest

The authors declare that they have no known competing financial interests or personal relationships that could have appeared to influence the work reported in this paper.
